# Outcomes of professional development to support capacity to provide eating disorder treatment and exploration of service level barriers

**DOI:** 10.1186/s40337-025-01308-9

**Published:** 2025-06-18

**Authors:** Emma C. Spiel, Rebecca Barns, Gabriella A. Heruc, Kim Hurst, Sarah Trobe, Siân A. McLean

**Affiliations:** 1 National Eating Disorders Collaboration, Melbourne, Australia; 2 Translational Health Research Institute and School of Medicine, Western Sydney University, Sydney, Australia; 3 Australia & New Zealand Academy for Eating Disorders, Sydney, Australia; 4 Robina Private Hospital, Gold Coast, Australia; 5 School of Psychology and Public Health, La Trobe University, Melbourne, 3083 Australia

**Keywords:** Eating disorders, Professional development, Training, Supervision, Barriers, Dietitian, Mental health clinician, Treatment, Workforce, Credential

## Abstract

**Background:**

The prevalence of eating disorders is increasing, with substantial impacts upon the person with the eating disorder, families, supports, and communities, as well as broad social and economic impacts. Most people who have an eating disorder either do not receive treatment, or experience substantial delay between symptom onset and treatment. To address the increasing prevalence and widespread impacts of eating disorders, both effective *and* accessible treatment interventions are required. There has been considerable focus on developing effective treatment interventions for eating disorders, but less attention has been given to increasing provision of treatment. This study reports on the impact of professional development for clinicians in increasing capacity to provide eating disorder treatment and perceived organisational barriers to doing so.

**Methods:**

Australian mental health professionals and dietitians (*N* = 397) completed an online survey exploring perceived level of skill, knowledge and willingness to provide safe, effective treatment for people experiencing eating disorders before and after completing one of three sequences of training and/or supervision, allocated based on their prior training and experience in eating disorder treatment. Participants also reported on perceived organisational barriers to the provision of eating disorder treatment.

**Results:**

Participants reported significantly higher levels of knowledge, skill, and willingness to treat eating disorders after receiving professional development than at baseline. There were no differences in the degree of skill and knowledge change based on the type of professional development received, however, the change in willingness to treat eating disorders after receiving professional development was higher among participants who received supervision only than among those who received introductory training, treatment model training and supervision. Lack of knowledge, skills, and abilities in staff and lack of funding were the most strongly endorsed barriers. Greater endorsement of barriers was associated with lower willingness to treat eating disorders at the end of the program.

**Conclusions:**

Low cost, large scale professional development in eating disorder treatment has the potential for widespread impact on workforce capacity and subsequent availability of evidence-based treatment. Further exploration to address the impact of organisational barriers on implementation is needed.

**Supplementary Information:**

The online version contains supplementary material available at 10.1186/s40337-025-01308-9.

## Abstract (plain language summary)

Eating disorders are a growing public health concern, and have significant psychological, medical, and physical harm, and high mortality rates. These disorders can impact individuals, families, and communities, with broad social and economic consequences. Despite increasing prevalence, access to effective treatment remains limited. This paper evaluates the impact of training and supervision delivered as part of professional development packages for clinicians in increasing their capacity to provide eating disorder treatment and explores barriers to doing so. Clinicians who took part in the professional development activities had higher levels of self-reported knowledge, skill, and willingness to provide treatment for people with eating disorders after they completed the training and or supervision. Structural barriers were associated with lower willingness to provide eating disorder treatment.

**Supplementary Information:**

The online version contains supplementary material available at 10.1186/s40337-025-01308-9.

## Outcomes of professional development to support capacity to provide eating disorder treatment and exploration of service level barriers

Eating disorders constitute a present and growing major public health concern [1, 2] and are associated with significant psychological, medical, and physical harm, conferring high suicide and mortality risk relative to other mental illnesses [3, 4]. Eating disorders significantly affect the quality of life of the person with the eating disorder [2], their families, supports, and communities [5], with broad social and economic impacts [6, 7]. Furthermore, eating disorder prevalence is increasing [8], in part through an exacerbation brought about by COVID-19 [see 9]. To address the increasing prevalence and widespread impacts of eating disorders, both effective *and* accessible treatment interventions are required [10]. While there has been considerable focus on developing effective treatment interventions for eating disorders [see 11], less attention has been given to increasing provision of treatment [12] despite the unmet need for treatment being well known [13]. The current paper will address this gap and evaluate the impact of professional development for clinicians in increasing capacity to provide eating disorder treatment. Barriers to increasing this capacity will also be explored.

Access to and uptake of timely, evidence-based treatment for eating disorders is influenced by multiple factors. Person-based factors that influence help-seeking are relatively well understood [e.g., 14], whereas factors related to the treatment context have been less extensively explored but may also play a significant role [15]. These include availability of a sufficiently skilled workforce, attitudes and capabilities of treating clinicians, organisational-level factors, and higher-level systems factors such as public policy and service funding [16, 17]. The potential impact of these factors is explored further below.

Mental health workforce shortages are a significant global issue [18, 19]. In Australia, the ageing workforce and rapid staff turnover due to stress, burnout, and lack of job security are considered key contributors to the mental health professional workforce shortage [19]. Staff shortages have also been reported in specialist eating disorder services. For example, in the United Kingdom, services were identified as being severely under-staffed, impacting the ability to meet demands and needs of existing service users and new referrals [20]. Inadequate training in eating disorder treatment provision in tertiary education, for example for medical practitioners and dietitians [21, 22] may compound the impact of workforce shortages across treatment settings on delivery of eating disorder services, as health professionals feel underprepared to work with eating disorders [23, 24].

Studies have consistently demonstrated a reluctance among clinicians to provide treatment and support for eating disorders, which can constrain the availability of treatment providers and contribute to delays in accessing care [e.g., 25, 26]. Among inexperienced health providers, desire to treat people with eating disorders or to specialise in this area is low [27, 28]. Similarly, medical and allied health practitioners report low levels of comfort and confidence to work with eating disorders [29, 30], however, greater comfort or confidence was reported by those who had at least some experience providing eating disorder treatment [29] or had received eating disorders training or clinical supervision [30]. Despite training being recognised as important, many practitioners, such as psychiatry residents [28] and dietitians [23], perceive that the teaching they had received about eating disorders in their degree programs had not been adequate.

Consistent with these findings regarding experience, attitudes of health professionals who specialise in eating disorders appear to be more favourable towards working in this area [31, 32]. While this may seem promising, relying solely on specialisation is limited as a pathway to increase capacity. It takes considerable time to gain the experience needed to become a specialist, and only a limited number of health professionals are likely to specialise in eating disorders. Therefore, a more promising avenue to increase capacity for working with eating disorders may be to provide additional post-degree training and professional development support for health professionals working across settings, including in non-specialist services [30]. This approach would not only increase capacity but also improve access to competent care by dispersing it beyond specialist services.

While interventions that deliver training can target clinician attitude change and skill development in eating disorder treatment, research evaluating their outcomes is limited [33]. In the available literature, positive outcomes of training include improved confidence, knowledge, and skill [e.g., 34, 35–37]. These are important outcomes as clinician self-efficacy is related to self-reported fidelity to delivery of evidence-based treatment [38]. Willingness to work with eating disorder clients has seldom been explored as a training outcome, with two studies showing little to no change in willingness after completion of training [34, 39]. The authors suggested this may reflect ceiling effects, where willingness to treat eating disorders was high prior to receiving training.

A review of current evidence suggests that interactive training, including skill demonstration and practice opportunities, is more effective than didactic, passive approaches in improving knowledge and changing therapist practices [40]. Furthermore, training alone has been found to be insufficient to drive sustained practice change without addressing key implementation factors. These can include ongoing support such as expert consultation, clinical supervision and feedback, or attention to structural factors that facilitate or impede implementation [40]. Clinical supervision has been identified as a necessary adjunct to improve outcomes from training [40, 41], including supporting skill development among psychology trainees [42] and promoting greater fidelity to evidence-based treatment [43]. In the context of eating disorders, practitioners have highlighted the benefits of ongoing supervision following training to support competence, evaluate practice, and reinforce knowledge acquired in training [44, 45]. In addition, evidence suggests that supervision can lead to equivalent clinical outcomes, even when treatment is delivered by trainee psychologists or non-specialised clinicians under the guidance of eating disorder experts [46, 47].

Following training, clinicians return to workplaces that can either foster or hinder the application of their new knowledge and skills as well as motivation to provide treatment. In the eating disorder field, organisational factors such as leadership support have been recognised as important for service delivery. For instance, managerial support is perceived as crucial for the adoption and provision of family-based therapy (FBT) and uptake of treatment for people with eating disorders [44, 45]. Managerial support is reflected by openness or ‘buy-in’ to the new approach and can also involve financial support for training attendance during work hours and access to ongoing supervision [45]. Adequate facilities and equipment also play a vital role in facilitating appropriate treatment delivery [44, 48].

At present, a challenge exists to build a workforce and a system of care that provides timely access to evidence-based treatment for eating disorders. We aimed to determine whether a large-scale training and supervision initiative, founded on evidence-based treatment implementation principles improved clinicians’ knowledge, skill, and willingness to provide treatment for people experiencing eating disorders. Additionally, for clinicians working in public health settings, we aimed to understand the organisational factors that were associated with greater change in clinician treatment provision capacity, to inform the effectiveness of future evidence-based treatment implementation initiatives. Specifically, we sought to address the following research questions:

1. What is the change in clinician self-perceived capacity to provide evidence-based treatment (knowledge, skill, willingness to treat eating disorders) following participation in a low-intensity professional development initiative?
2. Does change in self-perceived capacity differ across format of professional development received; (1) introduction to eating disorder training plus evidence-based treatment training plus supervision, (2) evidence-based treatment training plus supervision, and (3) supervision only?
3. What are the key organisational barriers to providing evidence-based treatment identified by clinicians in public health settings?
4. What is the relationship between organisational barriers and change in self-perceived capacity to provide evidence-based treatment following participation in the professional development initiative for clinicians working in public health settings?

## Method

### Participants

The current study reports on data from 397 participants from a larger study examining clinician engagement in a professional development initiative for eating disorders. In the larger study, 896 health professionals self-selected to be part of a workforce development initiative delivered by the National Eating Disorder Collaboration (NEDC). This offered free training and/or supervision to be able to meet criteria for the Australia & New Zealand Academy for Eating Disorders (ANZAED) Eating Disorder Credential [49] and were invited to take part in the research. Of these, *N* = 887 (99% of total participants) consented to the research and completed baseline surveys. The data for the present analyses are from participants who completed data collection at both baseline and following receipt of a professional development package (*n* = 397, 44.8% of research participants).

Healthcare professionals were eligible to receive professional development through this initiative if they were one of the professional groups eligible to become credentialed as a mental health professional (counsellors, general practitioners as providers of Focused Psychological Strategies, mental health nurses, nurse practitioners, occupational therapists, psychiatrists, psychologists, psychotherapists, and social workers) or as dietitians (see connected.anzaed.org.au).

### Measures

#### Demographic characteristics

Participants reported several personal and professional characteristics. These included age, gender, ethnicity, professional discipline (mental health professional vs. dietitian), state of residence, primary workplace type (eating disorder specific service, general mental health service or general health service), and years’ experience providing eating disorder treatment (do not treat eating disorders, 1–5 years, 6–10 years, 11–15 years, 16–25 years, or 25 + years).

#### Clinician knowledge, skill and willingness to provide treatment

A self-report measure was developed specifically for this study, based on prior research [50], to assess participant perceived level of skill, knowledge, and willingness to provide safe, effective treatment for people experiencing eating disorders. Responses were indicated on a 5-point Likert-type scale (1 = *very limited/not at all*, 2 = *limited/not very*, 3 = *average/somewhat*, 4 = *good/willing* 5 = *excellent/very willing*). At baseline, participants indicated their level of knowledge, skill, and willingness to treat each of body image concerns, binge eating disorder, bulimia nervosa, anorexia nervosa, and OSFED and responses were averaged to form a total score for each indicator with higher scores reflecting greater self-perceived knowledge, skill, and willingness. At post intervention, knowledge, skill, and willingness scores were calculated based on single item ratings of skill/knowledge/willingness to deliver safe and effective treatment for people experiencing eating disorders.

#### Organisational barriers

Organisational barriers to provision of eating disorder treatment were assessed with a scale developed for this research, based on constructs from the Consolidated Framework for Implementation Research, a conceptual framework that helps researchers and practitioners understand and assess factors that influence the implementation of complex interventions [51]. Participants rated 14 items, such as “Negative attitudes toward eating disorders” and “Lack of leadership support” as *not a barrier* (0), *minor barrier* (1), *moderate barrier* (2), or *major barrier* (3) in regard to the extent that they were barriers to providing safe, effective, and sustainable treatment to people experiencing eating disorders. To determine the factor structure of the organisational barriers measure, exploratory principal components analysis (PCA) was conducted using baseline data from the full sample from the larger study (*N* = 887). Oblique rotation (Oblimin) was used to aid interpretation. The PCA revealed three components with eigenvalues greater than one. A parallel analysis with Monte Carlo simulation (1,000 randomly generated samples of 12 variables x 887 participants) provided support to retain three factors for which 65.41% of variance was explained. All items were retained with no factor loadings < 0.4 and no items loading on more than one component following rotation. Scores for subscales identified through PCA were calculated from the mean of participant responses (range 0 to 3) with higher scores reflecting greater endorsement of workplace barriers to provision of eating disorder treatment. Subscales were named: structural barriers (6 items), which reflects lack of administrative support or structure to provide eating disorder treatment; culture barriers (4 items) which reflects attitudes towards eating disorders of colleagues and management; and resource barriers (4 items) which reflects lack of time, equipment, and funding to implement eating disorder treatment (see Table 1 for items). Internal consistency reliability in the current sample was adequate for all subscales (McDonald’s ω = 0.878, 0.814, and 0.831 for structural, culture, and resource barriers, respectively).

**Table 1:** Means and standard deviations for organisational barriers to provision of safe, effective, and sustainable eating disorder treatment for the total sample and by workplace setting

	Total sample		Public health *n* = 244		Private practice *n* = 153	
Items	Mean	*SD*	Mean	*SD*	Mean	*SD*
Structural barriers items						
Lack of required knowledge, skills, and abilities in staff ^a^	1.47	1.05	1.92	0.94	1.19	1.01
No/poorly articulated policies, procedures or guidelines to support eating disorder treatment ^a^	1.15	1.03	1.4	1.02	0.99	1.01
Lack of role clarity within the workplace for treatment of eating disorders ^a^	0.89	0.98	1.33	1.03	0.61	0.83
Lack of clear referral pathways ^a^	1.02	0.99	1.25	1.06	0.88	0.92
Lack of leadership support ^a^	0.89	1.01	1.11	1.06	0.76	0.95
Lack of access to appropriate training/supervision ^a^	1.27	1.08	1.50	1.13	1.12	1.02
Cultural barriers						
Organisational culture ^b^	0.55	0.83	0.90	0.94	0.32	0.67
Organisation perceives eating disorders as too specialised/out of scope ^b^	0.70	1.00	1.28	1.13	0.34	0.69
Negative attitudes towards eating disorders ^b^	0.38	0.75	0.69	0.92	0.18	0.54
Lack of interest in eating disorders of other clinicians ^b^	0.62	0.85	0.88	0.93	0.45	0.76
Resources barriers						
Inadequate equipment/facilities (i.e., scales, rooms for families) ^c^	0.98	0.98	1.29	1.04	0.78	0.90
Insufficient time to implement and deliver evidence-based treatment ^c^	1.06	1.05	1.48	1.09	0.80	0.93
Lack of time for care coordination ^c^	1.21	1.05	1.42	1.04	1.07	1.04
Lack of funding to deliver appropriate treatment ^c^	1.31	1.12	1.72	1.10	1.06	1.07
Subscales						
Structural	1.11	0.81	1.42	0.78	0.92	0.76
Culture	0.56	0.69	0.94	0.76	0.33	0.52
Resources	1.14	0.85	1.48	0.87	0.93	0.77

Note. Response options were 0 (not a barrier), 1 (minor barrier), 2 (moderator barriers), or 3 (major barrier); ^a^ items from structural subscale; ^b^ items from culture subscale; ^c^ items from resources subscale

### Professional development packages

For the professional development package intervention, depending on prior learning and experience, access was provided to introductory eating disorders training, and/or treatment model training, and/or supervision (3 group sessions and 3 individual sessions). Treatment model training was in either cognitive behavioural therapy-enhanced (CBT-E), FBT, cognitive behavioural therapy-guided self-help (CBT-GSH) or specialist supportive clinical management (SSCM) depending on preference and service role for mental health professionals, and evidence-informed dietetic practice for dietitians. Training packages were delivered online in small groups, were of five hours duration for introductory training and 5–16 h duration for treatment model training, and had been approved as meeting criteria for the ANZAED Eating Disorder Credential, set out in the National Framework for Eating Disorders Training [52]. Supervision was delivered by a clinician who meets the ANZAED Eating Disorder Credential criteria, has over 2 years clinical experience providing treatment for people experiencing an eating disorder, and over 2 years’ experience providing clinical supervision and/or has completed endorsed supervision training.

Participants were assigned to one of the three professional development packages based on the level of evidence-based training they had previously received. Package One provided introductory training plus treatment model/dietetic practice training and supervision sessions. Participants (*n* = 132) who identified as having had no introductory training in eating disorders nor in an evidence-based eating disorder treatment model (for mental health professionals) or dietetic practice (for dietitians) received this package. Package Two provided treatment model/dietetic practice training and supervision sessions. Participants (*n* = 180) who had completed introductory training in eating disorders but not an evidence-based eating disorder treatment/dietetic training received this package. Package Three provided supervision sessions only and was for participants (*n* = 85) who had previously completed both introductory and treatment model/dietetic practice training.

### Procedure

Ethics approval for this research was granted by the La Trobe University Human Research Ethics Committee (HREC Approval Number HEC22013). Clinicians completed evaluation surveys as part of their participation in the professional development packages. Research participation was voluntary. Participants gave written informed consent via an online survey completed prior to undertaking their allocated training and supervision program. Participation (or not) in the research did not affect participant access to the professional development program or relationship with the organisations involved in the program. Participants could withdraw consent at any time.

Demographic data, knowledge, skill, and willingness items, treatment provision items and organisational barriers scales were completed by all participants at baseline and again after completing the last component of their respective package.

## Results

### Participant characteristics

As shown in Table 2, most participants were cis-female, not of Aboriginal or Torres Strait Islander background, and spoke only English at home. Participants’ geographical location across states and territories in Australia reflected that of the general population and participants were relatively evenly distributed across age groups. In relation to professional characteristics, participants were mostly dietitians and psychologists. Years of experience working with eating disorders ranged from those who did not yet provide treatment through to those with decades of experience and the distribution of experience was in keeping with eligibility criteria for receiving the different professional development packages (see Table 3).

**Table 2:** Participant characteristics

		*n*	%
Profession	Counsellor	20	5.0
	Dietitian	155	39.0
	General Practitioner	8	2.0
	Mental Health Nurse	10	2.5
	Nurse Practitioner	1	0.3
	Occupational Therapist	4	1.0
	Psychiatrist	1	0.3
	Psychotherapist	3	0.8
	Psychologist	163	41.1
	Social Worker	32	8.1
Primary work setting	Private practice	244	61.5
	Public health or other	153	38.5
Age (years)	20–29	98	24.7
	30–39	139	35.0
	40–49	94	23.7
	≥ 50	66	16.6
Gender ^a^	Cisgender woman	371	93.5
	Cisgender man	24	6.0
	Non-binary / gender diverse	1	0.3
	Prefer not to say	1	0.3
Indigenous background ^a^	Aboriginal	4	1.0
	Neither Aboriginal nor Torres Strait Islander	393	99.0
Language spoken (in addition to English)	English only	339	85.4
	Italian	2	0.5
	Greek	2	0.5
	Cantonese	9	2.3
	Mandarin	10	2.5
	Arabic	5	1.3
	Vietnamese	3	0.8
	Other	31	7.8
Location (state/territory)	Australian Capital Territory	17	4.3
	New South Wales	118	29.7
	Northern Territory	5	1.3
	Queensland	78	19.6
	South Australia	20	5.0
	Tasmania	19	4.8
	Victoria	103	25.9
	Western Australia	37	9.3

Note. ^a^ other response options were available but no responses were received

**Table 3:** Years of experience providing eating disorder treatment according to professional development package type

		Years of experience, *n* (%^a^)					
	Total sample, *n* (%^b^)	0^c^	1–5	6–10	11–15	16–25	> 25
Package 1	132 (33.2)	57 (43.2)	59 (44.7)	10 (7.6)	4 (3.0)	2 (1.5)	0 (0)
Package 2	180(45.3)	21 (11.7)	130 (72.2)	14 (7.8)	7 (3.9)	6 (1.1)	2 (1.1)
Package 2	85 (21.4)	3 (3.5)	50 (58.8)	14 (16.5)	9 (10.6)	6 (7.1)	3 (3.5)

Note. ^a^ percent within package type; ^b^ percent within total sample; ^c^ do not yet provide eating disorder treatment

### Change in clinician self-perceived capacity to provide evidence-based treatment

Levels of knowledge, skill, and willingness to provide eating disorder treatment at baseline and Time 2 for the total sample and across professional development package type and participant profession are shown in Table 4. Comparison of self-reported Time-2 levels of knowledge and skill relative to baseline levels among the total sample was conducted with paired-samples t-tests. Significantly higher levels of knowledge, *t*(396) = 24.03, *p* <.001, *d* = 0.83 and skill, *t*(396) = 24.03, *p* <.001, *d* = 0.86, were found after receiving professional development than at baseline. Effects were large. Wilcoxon’s signed rank test (for non-normally distributed data) found willingness to provide eating disorders treatment was significantly higher at Time 2 relative to baseline in the total sample, with small effect size, *z* = -4.22, *p* <.001,|r| = − 0.21.

**Table 4:** Means and standard deviations for knowledge, skill, and willingness to provide eating disorder treatment according to professional development package type and profession

	Baseline M (SD)			Time 2 M (SD)		
	Mental health professionals	Dietitians	Total	Mental health professionals	Dietitians	Total
Knowledge						
Package 1	2.60 (0.77)	^a^	2.62 (0.77)	3.90 (0.63)	^a^	3.90 (0.52)
Package 2	3.17 (0.77)	2.87 (0.79)	3.06 (0.79)	4.13 (0.60)	4.12 (0.55)	4.11 (0.55)
Package 3	3.60 (0.66)	3.89 (0.62)	3.71 (0.65)	4.10 (0.45)	4.27 (0.57)	4.16 (0.51)
Total	2.96 (0.85)	3.20 (0.83)	3.05 (0.85)	4.00 (0.54)	4.14 (0.54)	4.06 (0.54)
Skill						
Package 1	2.08 (0.78)	^a^	2.10 (0.80)	3.66 (0.63)	^a^	3.67 (0.63)
Package 2	2.93 (0.79)	2.71 (0.97)	2.78 (0.92)	3.93 (0.61)	3.91 (0.58)	3.92 (0.59)
Package 3	3.42 (0.76)	3.77 (0.68)	3.56 (0.75)	3.98 (0.46)	4.12 (0.65)	4.04 (0.54)
Total	2.58 (0.96)	2.94 (1.01)	2.72 (1.00)	3.80 (0.61)	3.95 (0.60)	3.86 (0.61)
Willingness						
Package 1	3.97 (1.06)	^a^	3.99 (1.05)	4.20 (0.77)	^a^	4.20 (0.77)
Package 2	4.17 (0.82)	4.17 (0.90)	4.17 (0.87)	4.37 (0.69)	4.39 (0.68)	4.38 (0.68)
Package 3	4.39 (0.59)	4.55 (0.58)	4.45 (0.59)	4.46 (0.70)	4.76 (0.44)	4.58 (0.62)
Total	4.11 (0.93)	4.26 (0.85)	4.17 (0.90)	4.30 (0.74)	4.47 (0.65)	4.37 (0.71)

Note. Package 1 *n* = 132; Package 2, *n* = 180; Package 3, *n* = 85; ^a^data not provided where cell size < 5

**Fig. 1:**
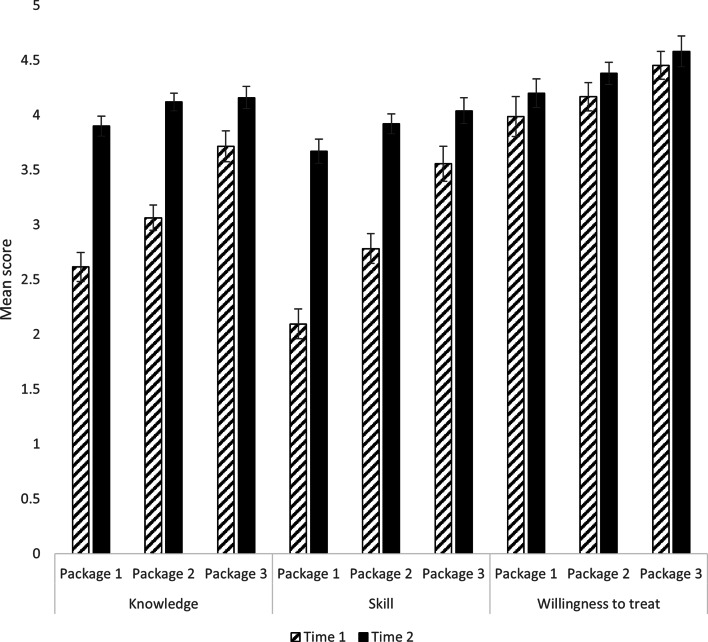
Baseline and Time 2 Means and 95% Confidence Intervals for Knowledge, Skill, and Willingness to Treat Eating Disorders for Participants in each of the Professional Development Packages. Note. Error bars are 95% confidence intervals. Means are unadjusted

Analyses of covariance (ANCOVA) were used to compare time two levels of knowledge and skill between participant groups receiving the three professional package types, controlling for baseline levels of knowledge and skill, respectively. These confirmed that there were no significant differences between professional package groups in knowledge, *F*(2, 393) = 2.26, *p* =.105, η_p_^2^ = 0.011 or skill, *F*(2, 393) = 1.94, *p* =.145, η_p_^2^ = 0.010 at Time 2, adjusting for baseline levels. Baseline and Time 2 mean scores are shown in Fig. 1 and adjusted means are shown in an additional file [see Additional file 1]. Due to non-normally distributed data, non-parametric analysis with Quade’s test was used to compare willingness to treat people with eating disorders at time two across professional development package types, controlling for Time 1 willingness. This confirmed a significant difference between groups on willingness at Time 2, *F*(2, 394) = 4.57, *p* =.011, η^2^ = 0.023. Scheffe post-hoc tests indicated that willingness to treat people with eating disorders was lower among participants who received professional development package 1 compared with those who received professional development package 3 (*p* =.012).

Due to unbalanced sample sizes of professions in different packages received, and dietitians receiving different training from mental health professionals, analyses comparing outcomes between professions were not conducted.

### Organisational barriers to provision of safe, effective, and sustainable eating disorder treatment

Organisational barriers to the provision of eating disorder treatment were not highly endorsed by participants. As shown in Table 1, in regard to individual items, most of the assessed aspects were, on average, perceived not to be a barrier to provision of treatment within participants’ workplaces. Given that organisational barriers have low relevance for clinicians in private practice, we provide descriptive statistics for these clinicians for exploratory purposes only. For clinicians working in public health settings, aspects of the workplace related to structures and resources were considered to be minor barriers. Lack of knowledge, skills, and abilities in staff and lack of funding to deliver appropriate treatment were the most strongly endorsed barriers. Average scores for endorsement of the cultural barriers subscale fell below the threshold to be considered a minor barrier.

Univariate linear regression analyses examined the prediction of Time 2 knowledge, skill, and willingness to treat eating disorders by structural, culture, and resources barriers. These analyses were conducted only with data from clinicians in public health settings (*n* = 153). Time 1 knowledge, skill, and willingness were included as covariates in respective analyses. In univariate analyses, none of the barriers predicted Time 2 knowledge, nor Time 2 skill levels. Time 2 willingness was predicted by each of the barriers subscales in univariate analyses, whereby greater structural, culture, and resource barriers were associated with lower willingness to treat eating disorders at Time 2, controlling for Time 1 willingness levels. See Table 5 for summary statistics.

**Table 5:** Summary statistics for univariate linear regression analyses predicting Post-Professional development package knowledge, skill, and willingness to treat eating disorders from organisational barriers for clinicians in public health settings

	b	95%CIs	*p*	*R* ^2^
Independent variable	Dependent variable: Knowledge			
Structural barriers	-0.011	-0.027, 0.004	0.156	0.12
Culture barriers	-0.004	-0.028, 0.020	0.756	0.11
Resources barriers	-0.018	-0.038, 0.003	0.094	0.13
	Dependent variable: Skill			
Structural barriers	-0.005	-0.022, 0.013	0.606	0.22
Culture barriers	0.012	-0.014, 0.037	0.376	0.22
Resources barriers	-0.011	-0.034, 0.012	0.345	0.22
	Dependent variable: Willingness to Treat			
Structural barriers	-0.042	-0.064, -0.019	< 0.001	0.20
Culture barriers	-0.036	-0.071, -0.000	0.047	0.15
Resources barriers	-0.044	-0.075, -0.013	0.005	0.17

Note. For brevity, the relevant Time 1 covariates are not included in the table

A follow up multivariable linear regression predicting Time 2 willingness to treat eating disorders was conducted. Each of the significant univariate predictors was included in the analysis, and Time 1 willingness was included as a covariate. In this multivariable analysis, 20.6% of variance was accounted for and structural barriers predicted Time 2 willingness, *b* = -0.039 [-0.033, -0.006], *p* =.019, whereby greater perception of structural barriers was associated with lower willingness to treat eating disorders at Time 2. Neither culture, *b* = 0.012 [-0.033, 0.057], *p* =.600, nor resource barriers, *b* = -0.016 [-0.055, 0.022], *p* =.403, predicted Time 2 willingness to treat eating disorders.

## Discussion

The aim of this study was to examine clinician self-perceived capability to provide eating disorder treatment following engagement in a professional development initiative. Participants who received one of a combination of (1) introductory training to eating disorders, (2) training in a treatment model or evidence-informed dietetic practice, and (3) supervision, had significantly higher eating disorder knowledge, skill, and willingness to provide safe and effective eating disorder treatment after receiving professional development relative to before. Change in these capabilities largely did not differ between groups after they had received professional development, with the exception of willingness to treat eating disorders. Willingness to treat people with eating disorders was higher in participants who had received supervision only than those who had received introductory training, treatment model training, and supervision, after controlling for Time 1 levels. The short-term nature of the post-intervention data collection, i.e., taking place shortly after completion of professional development, may account for these lack of group differences, particularly for willingness, as full effects of professional development may not emerge until after a period of engagement in practice has occurred.

Organisational barriers to providing safe and effective eating disorder treatment identified by participants were categorised as structure, culture, and resource related. Most participants indicated that organisational factors had only a minor, or lower impact as barriers to their ability to provide eating disorder treatment, regardless of their practice setting. Among clinicians in public health settings, structural, cultural, and resource barriers did not predict change in knowledge or skill, whereas each of the three barriers predicted change in willingness to treat eating disorders. Multivariable analyses revealed that of the three barrier subscales, only structural barriers predicted willingness, such that higher endorsement of structural barriers predicted lower willingness to treat eating disorders.

Clinicians who participated in professional development had large increases in their knowledge and skill for treating eating disorders, aligning with past research [34–37]. These findings confirm that professionals from various multidisciplinary backgrounds can enhance their eating disorder treatment capabilities through engaging in different levels of training and support, matched to clinician need. To our knowledge, this is the first study to have observed improvements in eating disorder treatment capabilities solely through clinical supervision [cf. 37], indicating the value of supervision. In addition, although participants in this study largely comprised psychologists and dietitians, medically trained health professionals also took part, with our findings adding to the relatively limited research on eating disorder training outcomes for these professionals. Similar to the current findings, recent research has demonstrated increases in self-reported capacity to provide eating disorder assessment and treatment among general practitioners who engaged in online microlearning [53] and increases in comfort in screening for and making eating disorder treatment referrals among paediatric primary care physicians and nurses who engaged with a one-hour asynchronous video training [54].

The present study also contributes a novel aspect by finding an increase in willingness to treat eating disorders following engagement with professional development. Although previous research has linked positive attitudes towards treatment to experience levels [32], past evaluations of professional development outcomes have yielded mixed results. Some studies have shown no change in willingness [34], while others have reported increases only for treating specific eating disorder subtypes [39]. The authors of these studies suggested that lack of change may be attributed to high baseline willingness to engage in provision of eating disorder treatment, as was observed here, which may also account for the small effect size. Interestingly, participants who received only supervision exhibited higher post-professional development willingness than those who received all three components, perhaps reflecting a shift for new clinicians (largely in the other professional package groups) in awareness of the breadth and depth of knowledge and skills required for effective treatment of eating disorders. In this manner, new awareness may have tempered pre-training willingness based on a more complete understanding of what is required for eating disorder treatment. For experienced clinicians, supervision may have reinforced existing knowledge, leading to increased motivation or reinvigorated practice.

Participants generally perceived organisational barriers to practicing eating disorder treatment as somewhat low. This might be due, in part, to clinicians who felt supported by their organisations to provide eating disorder treatment possibly being more likely to engage in this professional development. Additionally, organisational barriers may not yet have been apparent for those with limited or no experience treating eating disorders. Despite low levels of endorsement of barriers, these barriers were associated with specific treatment capacity outcomes. Notably, structural barriers was a significant predictor of lower willingness to treat eating disorders (multivariable analyses). These findings underscore the importance of structural factors such as administrative support in providing organisational systems or processes to support delivery of eating disorder treatment or facilitating access to appropriate training or supervision, in clinician’s perceptions of their capacity to treat eating disorders, as previously recognised [44, 45]. This suggests that aspects of organisational support such as role clarity, policies and procedures, and presence of others who are also skilled support clinicians in their individual work with eating disorders.

Several clinical implications arise from the present findings. The professional development initiatives described here are simple, cost-effective, and accessible interventions that can increase workforce capacity by expanding the number of clinicians equipped to provide appropriate, evidence-based eating disorder treatment. In turn, this increased workforce capacity can lead to better outcomes for people with eating disorders, as clinicians who are willing and able to provide such treatment will be more readily available, and the treatment offered is more likely to be evidence-based. The online delivery of the training and the supervision ensure that professional development can be implemented in regional, rural, or remote settings, facilitating scaling up of delivery by enabling clinicians in various locations to participate. Further exploration of the ongoing effect of increased capacity in perceptions of knowledge, skill, and willingness on actual practice change and increased provision of eating disorder treatment is needed to fully understand the effects of professional development on workforce capacity. Similarly, exploring the type of supervision associated with improved outcomes could contribute to continued refinement of professional development.

Addressing structural barriers may enhance clinicians’ ability to provide eating disorder treatment. Supervision and other supports that guide provision of treatment may be particularly efficient targets. Research from Australian training clinics indicates that pairing expert supervision with a well-defined protocol helps ensure competence in delivering therapy for individuals with eating disorders, leading to outcomes comparable to those achieved by experienced clinicians [47]. Improving role clarity, referral pathways, and leadership support, along with increased access to training and supervision are achievable and can be effective without requiring substantial resources. For example, more accessible and less expensive alternatives to traditional face-to-face clinical supervision that support, direct and guide evidence-based practice exist. These include clinical supervision exchange models whereby a worker from one organisation provides clinical supervision to staff of another organisation, and vice versa [55], and group supervision programs (i.e., ANZAED online consultation). A systematic review of telehealth models of supervision shows that online models are feasible, cost-effective and beneficial [56] and address substantial barriers to access faced by rural and remote health practitioners. Publicly-funded online case consultation services and eating disorder specific peer group supervision have been shown to enhance the capacity of health professionals to identify and treat emotional and psychological challenges and improve clinician confidence and ability to deliver evidence-based services [57–59]. Bolstering the capacity of existing eating disorder specific case consultation services would likely address some of the structural barriers to large scale rollouts of skills-to-practice translation following training. Implementing these models and approaches would be useful to consider for supporting eating disorder specific service and workforce development initiatives at scale.

Several limitations require consideration. The self-reported nature of the outcomes assessment may be subject to bias or demand characteristics. This may have affected the findings for willingness to treat eating disorders, whereby the observed small change may have reflected that the sample recruited for the study were keen to receive professional development in eating disorders to achieve eligibility for the Credential, and thus were relatively willing to provide treatment at the baseline point of the study. Exploring change in willingness to treat eating disorders in a sample that does not self-select for professional development may help to provide further understanding of the impact of professional development in this domain. In addition, the study did not assess the level of knowledge gain or application of knowledge following participation in professional development activities. Objective observation of treatment capability to deliver treatment would strengthen future research. The absence of a control group limits the ability to attribute changes in self-perceived capacity to the professional development intervention. Other factors may have contributed to these changes. Although participants reported their willingness to provide eating disorder treatment, data on actual treatment provision and the number of people treated was not collected, limiting the ability to determine if professional development leads to increased treatment provision uptake. Finally, the sample of consenting research participants was a subsample of those who received the professional development packages and may not fully reflect the experiences of all participants.

## Conclusion

This study demonstrated that a low cost, large scale model for professional development in eating disorder treatment improved self-reported knowledge, skills, and willingness to treat eating disorders among clinicians from diverse discipline backgrounds and with varying levels of prior experience. Structural barriers were the most prominent obstacle for increasing capacity for providing safe, effective, and sustainable treatment to people experiencing eating disorders. Professional development in eating disorders comprising training and supervision can help clinicians meet the eligibility criteria for the ANZAED Eating Disorder Credential. By scaling up online delivery, these initiatives have the potential for widespread impact on workforce capacity and subsequent availability of evidence-based treatment, in line with the aims of the Credential.

## Electronic supplementary material

Below is the link to the electronic supplementary material.

Supplementary Material 1

## Data Availability

The datasets used and/or analysed during the current study are available from the corresponding author upon reasonable request.

## Declarations

### About this supplement

This article has been published as part of Journal of Eating Disorders Volume 13 Supplement 1, 2025: The Australia & New Zealand Academy for Eating Disorders. The full contents of the supplement are available online at: https://jeatdisord.biomedcentral.com/articles/supplements/volume-13-supplement-1

## Abbreviations

ANZAED: Australia & New Zealand Academy for Eating Disorders
NEDC: National Eating Disorder Collaboration
