# Supplementary material for: Outcomes of professional development to support capacity to provide eating disorder treatment and exploration of service level barriers
Source: J Eat Disord. 2025 Jun 18;13(Suppl 1):116. doi: 10.1186/s40337-025-01308-9 (PMC12175328; doi:10.1186/s40337-025-01308-9)
Supplement: Supplementary file 1 — Supplementary Material 1 [file 40337_2025_1308_MOESM1_ESM.docx]

Additional File 1

**Table A.1**

*Means (SD) and Adjusted Means (SE) for Knowledge and Skill According to Professional Development Package Type*

|  | Package 1 (*n* = 132)  *M (SD/SE)* | Package 2 (*n* = 180)  *M (SD/SE)* | Package 3 (*n* = 85) *M (SD/SE)* |
| --- | --- | --- | --- |
| Knowledge |  |  |  |
| Baseline | 2.62 (0.77) | 3.06 (0.79) | 3.71 (0.65) |
| Time 2 | 3.90 (0.52) | 4.12 (0.55) | 4.16 (0.51) |
| Time 2 (adjusted) | 4.00 (0.05) | 4.11 (0.04) | 4.02 (006) |
| Skill |  |  |  |
| Baseline | 2.10 (0.80) | 2.78 (0.92) | 3.56 (0.75) |
| Time 2 | 3.67 (0.63) | 3.92 (0.59) | 4.04 (0.54) |
| Time 2 (adjusted) | 3.88 (0.05) | 3.90 (0.04) | 3.75 (0.06) |

*Note*. Baseline covariates for adjusted means were baseline knowledge mean = 3.05 and baseline skill mean = 2.72
